# Non-Equilibrium Thermodynamics of Micro Technologies

**DOI:** 10.3390/e21050501

**Published:** 2019-05-17

**Authors:** Mohsen Torabi, Nader Karimi, Mostafa Ghiaasiaan, Somchai Wongwises

**Affiliations:** 1 The George W. Woodruff School of Mechanical Engineering, Georgia Institute of Technology, Atlanta, GA 30332, USA; 2 School of Engineering, University of California, Merced, CA 95343, USA; 3 School of Engineering, University of Glasgow, Glasgow G12 8QQ, UK; 4 School of Computing and Engineering, Civil and Mechanical Engineering Department, University of Missouri-Kansas City, Kansas City, MO 64110, USA; 5 Department of Mechanical Engineering, King Mongkut’s University of Technology Thonburi, Bangkok 10140, Thailand

**Keywords:** entropy generation, micro thermofluidic systems, micro channels, miniaturized cryocoolers, micro reactors

## Abstract

This is the Editorial article summarizing the scope and contents of the Special Issue, Non-Equilibrium Thermodynamics of Micro Technologies.

## 1. Second Law of Thermodynamics and Its Importance in Microscale Systems

Advancement of manufacturing techniques has resulted in a significant reduction in the size of the individual components within many devices. This advancement has put forward micro and nanoscale devices instead of macroscale counterparts. Miniaturized thermofluidic systems are a subcategory of this renovation, and has recently gained significant attention. Applications of these systems in micro manufactured devices is growing steadily. Some emerging applications of micro thermofluidic systems include, microreactors [5,6], micro heat sinks [7,8], and micro cryocoolers [9,10], to name a few. The progresses in the design, development and application of micro thermofluidic systems have resulted in high demand for their heat transfer and thermodynamics analyses. Hence, small-scale thermofluidic systems have been the subject of both theoretical and experimental investigations [1,2,3,4].

Scholars have tried to investigate these systems for years from the heat transfer perspectives and so far a number of reviews have appeared in connection with these systems [2,11]. With regards to the first law of thermodynamics and its application to the performances of these systems, temperature simulation [12] and visualization [13], heat flux investigation and optimization [14,15], and partial or total design reconfigurations [16,17,18] have been performed.

Recently, micro thermal systems have gained attention regarding the second law’s performance and researchers have started to focus on investigating micro thermal systems from the entropy perspective [19,20]. The second law analysis is a quantitative approach to evaluate the efficiency of thermofluidic systems [21]. The second law analysis provides scholars a tool to minimize the irreversibility of the system, and hence results in more environmentally friendly systems [22]. Regarding small scale thermofluidic systems, the second law investigations range from entropy generation in micro-channels [20,21,22,23] and concentric micro-fin tube heat exchanger [24] to micro combustors [25,26] and micro reactors [27,28]. To perform the second law analysis, various effects and specifications of the system including, but not limited to, fluid flow, heat and mass transfer, internal heat generation and electromagnetic effects should be included within analyses. There are relatively large number of textbooks which covers the concept of the second law analysis in thermofluidic systems [21,29,30,31]. However, because of rapid development of small-scale systems, there is an urgent need to perform more investigations on more diverse applications such as microreactors, micro-scale thermoelectric coolers, and new geometries of microchannels. The main motivation for this special issue is to diversify the available literature regarding the second law of thermodynamics in microscale thermofluidic system by contributing nine articles.

## 2. The 9 Contributions Published in This Special Issue

This special issue consists of nine articles that report on investigations dealing with heat and mass transfer and thermodynamic analysis of thermofluidic systems [32,33,34,35,36,37,38,39,40]. Most of these investigations are analytic or numerical [32,33,34,35,36,37,38,39,40]. Two of the investigations include experiments [36,39]. The main theme of four articles in this special issue is optimization of microchannels from both energetic and entropic perspectives [34,36,39,40]. Other areas that have been explored include the analysis and optimization of microreactors [33], simulation and thermal analysis of micro circular Couette flow [37], and thermodynamic analysis of various-shaped micro-scale thermoelectric coolers [38]. Moreover, an interesting concept named *virtual entropy generation method* has been elaborated by Zhang et al. [35], which provides a path for the evaluation of leakage in channels using the concept of entropy generation.

Thermodynamic analysis of micro thermofluidic systems is an emerging area. With the advancement of manufacturing techniques and rapid growth of micro-fabricated devices, design and optimization of small-scale thermofluidic systems need more attention. This special issue is one step towards this goal, and it is hoped that it will inspire further investigations.
